# Generation of Photopolymerized Microparticles Based on PEGDA Using Microfluidic Devices. Part 1. Initial Gelation Time and Mechanical Properties of the Material

**DOI:** 10.3390/mi12030293

**Published:** 2021-03-10

**Authors:** José M. Acosta-Cuevas, José González-García, Mario García-Ramírez, Víctor H. Pérez-Luna, Erick Omar Cisneros-López, Rubén González-Nuñez, Orfil González-Reynoso

**Affiliations:** 1 Chemical Engineering Department, CUCEI, Universidad de Guadalajara, Blvd.M. García Barragán # 1451, Guadalajara C.P. 44430, Jalisco, Mexico; jose.acosta@alumnos.udg.mx (J.M.A.-C.); joseglezgar@yahoo.com.mx (J.G.-G.); erick.cisneros@academicos.udg.mx (E.O.C.-L.); rubens_glez@hotmail.com (R.G.-N.); 2 Electronics Department, CUCEI, Universidad de Guadalajara, Blvd.M. García Barragán # 1451, Guadalajara C.P. 44430, Jalisco, Mexico; mario.garcia@academicos.udg.mx; 3 Department of Chemical and Biological Engineering, Illinois Institute of Technology, 10 West 33rd Street, Chicago, IL 60616, USA; perezluna@iit.edu

**Keywords:** PEGDA Hydrogel, photopolymerization, microfluidic devices, initial gelation time

## Abstract

Photopolymerized microparticles are made of biocompatible hydrogels like Polyethylene Glycol Diacrylate (PEGDA) by using microfluidic devices are a good option for encapsulation, transport and retention of biological or toxic agents. Due to the different applications of these microparticles, it is important to investigate the formulation and the mechanical properties of the material of which they are made of. Therefore, in the present study, mechanical tests were carried out to determine the swelling, drying, soluble fraction, compression, cross-linking density ($M_{c}$) and mesh size (ξ) properties of different hydrogel formulations. Tests provided sufficient data to select the best formulation for the future generation of microparticles using microfluidic devices. The initial gelation times of the hydrogels formulations were estimated for their use in the photopolymerization process inside a microfluidic device. Obtained results showed a close relationship between the amount of PEGDA used in the hydrogel and its mechanical properties as well as its initial gelation time. Consequently, it is of considerable importance to know the mechanical properties of the hydrogels made in this research for their proper manipulation and application. On the other hand, the initial gelation time is crucial in photopolymerizable hydrogels and their use in continuous systems such as microfluidic devices.

## 1. Introduction

**Figure 1:**
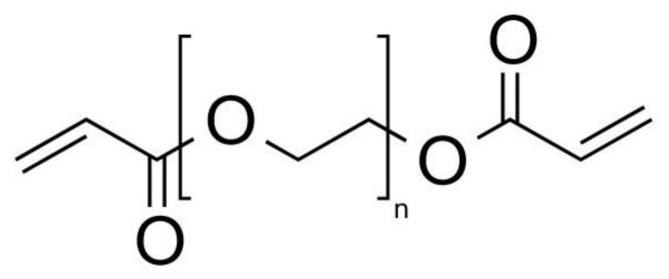
Representation of the Polyethylene Glycol Diacrylate (PEGDA) chemical structure in which *n* represents the ethylene glycol repetition unit and the extremes are acrylate groups.

As macroscopic processes in biology, chemistry and medicine have evolved to compact processes of microscopic scales, microparticles generation has been an attractive approach and a key factor in the evolution of these processes [1]. Microparticles generated by microfluidic devices have advantages of integrity, high efficiency, homogeneity in particle size and continuous production which represent a great potential for the biotechnological field [1,2,3]. The use of small volume in microparticles represents lower reagent costs, less waste generation and easy manipulation of the microparticle [4]. Generally, the generation of microparticles based on polymers such as hydrogels made of Polyethylene Glycol Diacrylate (PEGDA), Figure 1, are extensively used in the biotechnological field due to their minimal toxicity [5]. Different application fields have used hydrogels, such as biomedical devices [6,7,8,9,10], intelligent robotics [11,12], flexible electronics [13], mechanical metamaterials [14,15,16,17], activators [18,19] and the encapsulation of medicaments or biological agents where the hydrogel can be useful in protecting them or releasing them [20].

**Figure 2:**
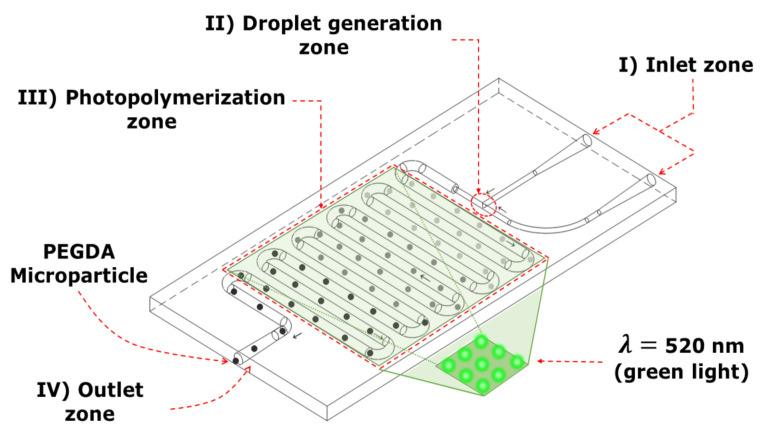
Design of a microfluidic device with four zones. (I) Inlet zone, (II) droplet generation zone, (III) photopolymerization zone and (IV) outlet zone. Green LEDs are used for the photopolymerization process. The microfluidic device design was made of poly-dimethyl siloxane (PDMS, Syl- gard 184, Dow Corning) [25].

There are different ways to produce hydrogel microparticles using microfluidic devices [21,22,23]. Xue et al. reported an in situ UV photopolymerization method using a microfluidic device to produce 5-fluorouracil loaded biocompatible PEGDA microspheres for drug release [24]. However, the formulation of the hydrogel’s material represents an important factor in the design of these microfluidic devices. The formulation of the hydrogel’s material can determine the residence time of microparticles and in turn the length of the photopolymerization zone in the microfluidic device; Figure 2. The importance of this zone is based on the formation of completely photopolymerized hydrogel droplets once they stop being exposed to visible light (λ≈ 520 nm).

Figure 2 shows the four zones of a microfluidic device. The inlet zone is where the immiscible liquid phases are injected. The droplet generation zone is the region where both phases will be mixed to obtain the hydrogel. The photopolymerization process has the longer zone in which the hydrogel droplet will be reached by green light. The exit zone is where the microparticles leave the microfluidic device. Furthermore, studies on droplet formation under different geometries have been already published [25].

**Figure 3:**
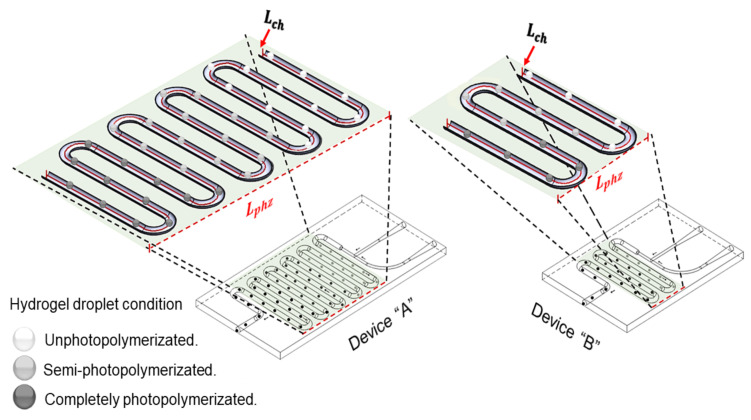
The initial gelation time may be different for different hydrogel formulations. $L_{ch}$ represents the total channel length required to reach the initial gelation point of the hydrogel. The shorter the length of the channel ($L_{ch}$), the smaller the length of the photopolymerization zone ($L_{phz}$).

In Figure 3 is presented the relation between the hydrogel formulation and the design of the microfludic device. The length of the photopolymerization zone ($L_{phz}$) depends on the length of the channel ($L_{ch}$) through which the droplet is transported. The smaller the size of the photopolymerization zone, the smaller the size of the microfluidic device. To reduce the length of the photopolymerization zone, the length of the channel or the injection speed of the immiscible liquid phases must be reduced. However, another option is to work with a hydrogel formulation with a short initial gelation time. A short initial gelation time could help to use higher injection speeds and thus generate a fast and continuous production of hydrogel droplets in microfluidic devices. As shown in Figure 3, the devices *A* represents a slow photopolymerization process with a long channel length, representing a hydrogel with a long initial gelation time. While in the devices *B* a fast initial gelation time in hydrogel will require a shorter channel length. A hydrogel formulation that starts the photopolymerization with a short initial gelation time will be desirable to reduce the total length of the channels ($L_{ch}$) in the photopolymerization zone of the microfluidic device as it can be seen in Figure 3. On the other hand, the hydrogel formulation that presents the shortest initial gelation time could demonstrate different mechanical properties compared with other hydrogel formulations.

Moreover, an important aspect when referring to hydrogel formulations is the cross-linking structure of it. The cross linked structure of a hydrogel shows two important properties called average number of molecular weight between two consecutive cross-links ($M_{c}$) and mesh size (ξ). These two properties influence the mechanical strength and swelling capacity of the hydrogel. The cross-linked polymer network of the hydrogel can be rigid, but due to its swelling capacity it can also become flexible [26,27,28]. The water contained in the hydrogels is qualified as free, semi-linked and linked which influences the mechanical properties of hydrogels making them tougher or more flexible [29,30,31,32]. Due to these properties, hydrogels show great potential in their application with microfluidic devices. Examples are Lab-on-a-chip devices [3,33], or platforms with dynamic motion and shape memory [34].

This study worked with Polyethylene Glycol Diacrylate 575 (PEGDA 575) in different mixtures varying its concentration and exposing it to visible light for the beginning of the photopolymerization process. It should be noted that no studies have been reported on the initial gelation time for hydrogels and their use in microfluidic devices. Therefore, we investigated the initial gelation time of each hydrogel formulation, its mechanical (compression), swelling and waste properties (absorption and drying) in order to find the best formulation with the shortest initial gelation time to obtain a suitable hydrogel for the future continuous production of microparticles using microfluidic devices. The wide development in the application of hydrogels shows the importance of also knowing the mechanical properties that they can offer us. We investigate the initial gelation time of different hydrogel formulations as well as their mechanical properties to contribute in the future generation of photopolymerized PEGDA microparticles using microfluidic devices.

## 2. Materials

Polyethylene Glycol Diacrylate (PEGDA) with an average molecular weight number (Mn) of 575 was used as the cross-linking agent due to its ramified diacrylates. Triethanolamine with a purity of 90% was added as co-initiator. As an accelerator 1-Vinyl-2-pyrrolidinone with a purity of 99% was used. The photoinitiator (PI), Eosin Y, was used with a purity of 99%. All the materials mentioned before were purchased from Sigma-Aldrich, St. Louis, MO, USA.

All reagents were used as they were received, without any additional modifications. Distilled water was used as a solvent for each formulation. Glacial acetic acid and ethyl alcohol were used with a purity of 99% and 96%, respectively. A Vortex Mixer was used to mix the solutions homogeneously. The molds required for the compression test were generated on a Dremel 3D printer model 20 (Dremel, Mt. Prospect, IL, USA). Molds were fabricated following the ASTM F2900 NORM specifications. The initial gelation times were measured with a Brookfield model DV-I Prime viscometer (Brookfield, New York, NY, USA).

### 2.1. Preparation of Eosin “Y” Solution

Several studies have used UV light to trigger photopolymerization adding an agent called Irgacure 2959 [35,36,37,38,39]. However, in this study, eosin “Y” was implemented as the precursor to photopolymerization, also called photoinitiator (PI), by using visible light (λ≈520 nm). Furthermore, eosin “Y” presents a good water solubility and a wide range of absorbance. Eosin “Y” solution was used for the hydrogel preparation with a concentration of 0.5 mM in ethyl alcohol which was employed as solvent. Two drops of glacial acetic acid were added as pH buffer to establish a pH value between 4 and 5. With respect to previous studies [40], the eosin “Y” concentration was lower in our research because of the lower molecular weight used of PEGDA.

### 2.2. Hydrogel Solution Synthesis

Gregory M. Cruise 1998 reported a good standard hydrogel solution with 25% (wt PEGDA/wt total solution). Here in this research, the above formulation was replicated, and we called it H-3; Table 1. However, to establish a better initial gelation time and to allow a good photopolymerization process, different hydrogel solutions were tested and the composition of the hydrogel solution was changed; Table 1. The solutions were gauged/calibrated in a 500 mL volumetric flask at a temperature of 25 °C. Firstly, a water bed was poured and then triethanolamine (225 mM), 1-vinyl pyrrolidone (37 mM) and PEGDA were added according to the quantities shown in Table 1. Eosin “Y” (0.5 mM) was added at the end of the solution preparation due to its photosensitive nature. Immediately, the mixture was calibrated and homogenized in a vortex mixer with a velocity between 2000 to 3000 rpm during 2 min. Volumetric flask was shielded to avoid contact with light.

### 2.3. Initial Gelation Time

**Figure 4:**
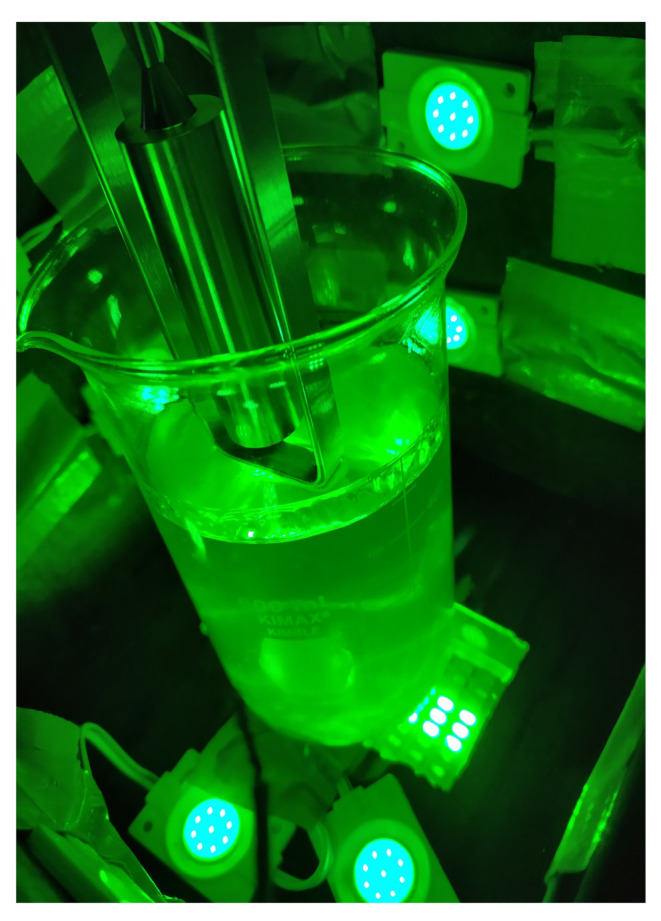
The 12 LEDs were coupled around the closed box while the LED lamp was installed under it. This closed box was then coupled to the DV-I Prime viscometer.

Characterization of the solutions with respect to the initial gelation time takes a very important role in the generation of photopolymerized microparticles using microfluidic devices. Even though a short initial gelation time could be achieved by changing the temperature [41], in the present study all the experiments were made at room temperature (25 °C). Another important element is the pH in the solution because it decreases the ion concentration which is important in the polymerization process [42]; however, the value of pH 8.5 remained constant for all the experiments.Therefore, experiments to estimate the initial gelation time of the different formulations were carried out at 500 mL; Table 1, with a initial pH of 8.5 and a temperature of 25 °C. These experiments were prepared by coupling the different solutions (Table 1) to a closed box in which there was a system of lamps that generated the necessary light for the beginning of the photopolymerization process. This system of lamps consisted of 12 LEDs, which were coupled around the box; each of them had a power of 2.4 W, and the total irradiated luminosity by them was 3.7 $\times \;10^{4}$ lx. Moreover, an additional LED lamp was installed under this closed box, which had a power of 50 W and a total irradiated luminosity of 6.6 $\times \;10^{5}$ lx. This closed box was coupled to the viscometer with the purpose of ensuring a uniform light incidence without any external interference. Once the viscometer was started up, the system of LED lamps was turned on and the viscometer showed the increment in the viscosity of the solution with respect to time. In Figure 4 this closed box is shown coupled to the viscometer. Three repetitions of each sample (H-1, H-2, H-3, H-4 and H-5) were performed to obtain an average of each initial gelation time.

On the other hand, for mechanical tests, the solution was calibrated in a 10 mL volumetric flask at a temperature of 25 °C. The reagents concentrations were the same as the described above: triethanolamine (225 mM), 1-vinyl pyrrolidone (37 mM) and eosin “Y” (0.5 mM). The volume used for each component is shown in Table 2.

### 2.4. Sample Preparation

**Figure 5:**
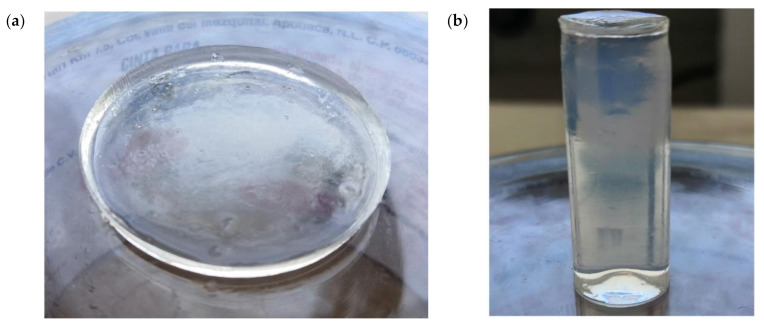
(**a**) Samples of PEGDA used in the swelling tests. (**b**) Samples of PEGDA used in the compression tests.

Plastic Petri dishes were used as molds for the swelling tests. The dimensions of Petri dishes were 35 mm diameter and 10 mm high. Each of the plastic dishes was poured with 2 mL of PEGDA solution. To generate the necessary samples used in the compression tests, a cylindrical mold was printed on the Dremel machine with a 12.7 mm of diameter and 25.4 mm high. PEGDA solution inside the molds was then directly exposed to green light (visible light) with approximately wavelength of 520 nm. Direct exposure time was 10 and 15 min for swelling and compression samples, respectively; Figure 5. All measurements used to obtain the samples were taken based on ASTM F2900 standards for hydrogel characterization.

### 2.5. Swelling Test

Swelling tests were carried out with distilled water. For each synthesized hydrogel formulation, five samples of each of them were immersed in distilled water for 24 h. Subsequently, each hydrogel sample was weighed and then dried on the Sartorius model MA35 electronic moisture analyzer at a temperature of 160 °C for 20 min. Once the first dry weights of each hydrogel sample were obtained, they were completely immersed in distilled water again to begin the second swelling process for other 24 h. Finally, the samples were dried again in the moisture analyzer for other 20 min and weighed to determine their second dry weight, after a second swelling. Swelling ratio *Q* (Equation (1)) and soluble fraction *F* (Equation (2)) were calculated with the following equations [43]:

(1) $$\begin{array}{c} Q=\frac{W_{s}-W_{i}}{W_{i}} \end{array}$$

(2) $$\begin{array}{c} F=\frac{W_{i}-W_{d}}{W_{i}} \end{array}$$

where $W_{s}$ represents the weight of hydrogel samples after first swelling, $W_{i}$ is the weight of dried hydrogel samples after first drying, and $W_{d}$ represents the weight of dried hydrogel samples after second drying. Swelling ratio *Q* [1] is defined as the fractional increase in hydrogel weight due to water absorption. In addition, it is possible to find the hydrogel degradation with respect to its swelling since the hydrogel has a soluble fraction *F* (2) that represents the polymer that continues in the reticulation reaction, and it is not part of the hydrogel. Two equations were used to perform the calculations.

For the 5 samples of each formulation, an average of the swelling ratio and the soluble fraction was calculated. The swelling ratio helps to quantify the volume fraction of the polymer, $V_{2,s}$, when the hydrogel is swollen. Knowing the density of water ($\rho_{H_{2}O}$ = 1.0 g/cm${}^{3}$) and PEGDA polymer (ρ=1.12 g/cm${}^{3}$), we can use the Equation (3).

(3) $$V_{2,s}=\frac{1}{Q\frac{\rho }{\rho_{H_{2}O}}+1}$$

The Peppas–Merrill equation (Equation (4)) allows the estimation of the average number of molecular weight between two consecutive cross-links, Mc, for the same type of cross-linked network (for the same hydrogel). Estimating the value of Mc it is possible to understand certain hydrogel swelling behaviors, (Equation (4)) [44,45].

(4) $$\frac{1}{M_{c}}=\frac{2}{M_{n}}-\frac{ln\left(1-V_{2,s}\right)+V_{2,s}+\chi {V_{2,s}}^{2}}{\rho V_{1}V_{2,r}{\left(\frac{V_{2,s}}{V_{2,r}}\right)}^{\frac{1}{3}}-\frac{1}{2}\left(\frac{V_{2,s}}{V_{2,r}}\right)}$$

where χ is the Flory–Huggins polymer/solvent dimensionless interaction parameter, which is assumed to be 0.426 according to the determination by [44]. $V_{1}$ is the molar volume of water (18 cm${}^{3}$/mol), and $V_{2,r}$ is the volume fraction of polymer in the hydrogel before swelling. Two equations described by Cavallo (Equations (5) and (6)) were used to estimate the value of the mesh size ξ.

(5) $${\left({r_{0}}^{-2}\right)}^{\frac{1}{2}}=l{\left(2\frac{M_{c}}{M_{r}}\right)}^{\frac{1}{2}}{C_{n}}^{\frac{1}{2}}$$

(6) $$\xi ={\left({r_{0}}^{-2}\right)}^{\frac{1}{2}}{V_{2,s}}^{\frac{-1}{3}}$$

where ${\left({r_{0}}^{-2}\right)}^{\frac{1}{2}}$ is the average end-to-end distance between adjacent bonds of two PEGDA repeat units; *l* is the bond length for PEGDA (1.5 Å); $M_{r}$ is the weight of the PEGDA repeat unit (44 g/mol) while $C_{n}$ is defined as the characteristic radius for PEGDA with a value of 4 [45].

### 2.6. Compression Tests

**Figure 6:**
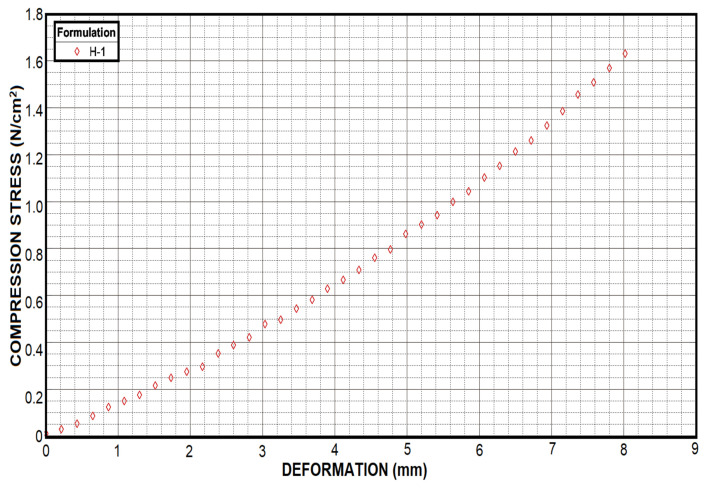
Even if the H-1 formulation does not have a resistance as high as those shown by the other formulations, it was able to double the deformation presented by H-3 and H-5, which translates into greater flexibility.

Compression tests were measured on hydrogel with the universal mechanical testing machine INSTRON 3345. The force applied for the tests was 5 kN. The deformation test was measured by the hydrogel compression between plates of the testing machine at a speed of 1.3 ± 0.3 mm/min. Five recently cross-linked hydrogels and swollen samples were coupled to the equipment, and the tests were performed at room temperature (25 °C) following ASTM F2900 standards for hydrogel characterization. One of the desired characteristics of hydrogel microparticles is the resistance to pressure and deformation that can be caused in a transport flow or in their field of use. The importance of obtaining a good resistance becomes evident by providing a longer lifetime and will ensure a good performance in a range of established pressures for the hydrogel. Additionally, it is possible to establish the flow parameters in microfluidic systems according to the resistance or deformation shown by the cross-linked hydrogel. To calculate the yield stress, the initial size of the cylindrical samples was compared to the final size after the compression test. The results showed a deformation degree that the hydrogel was capable of supporting before it broke (Equation (7)).

(7) $$\epsilon =\frac{\Delta L}{L_{i}}\times 100$$

where ΔL is the change in length, initial length $L_{i}$ minus final length $L_{f}$. Length $L_{i}$ was one inch while $L_{f}$ could be calculated with ΔL value reported in Figure 6.

### 2.7. Young’s Module

The Young’s module was calculated for each of the cylindrical samples in swollen state in the compression test with the INSTRON 3345 instrument. All samples were kept in a saturated moisture environment until testing. Young’s modulus, stress and unit deformation were calculated using the instrument and the Equation (8). In order to choose a good formulation focused on the formation of a microparticle, it is very important to know the measurements of strength and rigidity of the hydrogel. Thus, with the help of Young’s module calculation, it will be possible to classify the formulation according to a specific approach and application.

(8) $$E=\frac{\sigma }{\epsilon }$$

## 3. Results and Discussion

### 3.1. Formulation

**Figure 7:**
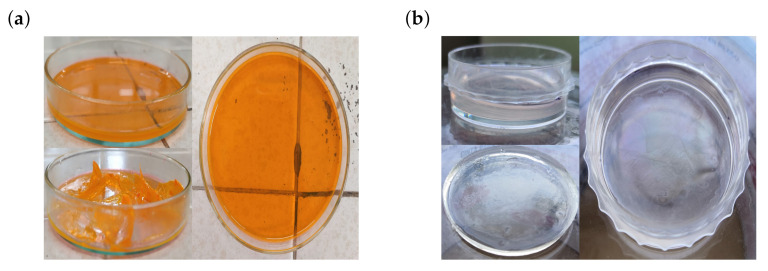
Hydrogel synthesis. (**a**) Using a concentration of 2 mM of Eosin “Y”. (**b**) Using a concentration of 0.5 mM of Eosin “Y”. It is significant that with a higher concentration of Eosin “Y”, a more colorful sample is produced.

Visual properties are an important aspect in the generation of hydrogels as is the color of them. In this paper specifically, when more PI is used in the PEGDA formulations, more colorfulness is obtained. Therefore, experimentation was carried out to obtain a translucent hydrogel which could be used in the encapsulation of photosynthetic microorganisms (Figure 7a). High coloration presented in the hydrogel influences the absorbance and transmittance of it. A high concentration of eosin “Y” will only lead to a recession in the photopolymerization process [16] and an increment in the initial gelation time. Therefore, it was decided to work with a low concentration of eosin “Y” in the formulations analyzed, showing better results in the coloration and cure of the hydrogel (Figure 7b).

Eosin “Y” has been proved to be a good photoinitiator at low concentrations although its use implies a co-initiator for a greater efficiency. Nevertheless, eosin “Y” has good solubility, low cytotoxicity and wide range of absorbance. The use of eosin “Y” represents also the possibility to use different light sources in the visible range and to use low light powers. However, coloration provided by eosin “Y” can become a problem if it is desired that the hydrogel allows penetration of light through it. A low concentration of the eosin “Y” (PI) will cause a pink coloring at first sight but the hydrogel will end up being clear.

The formulation of the hydrogels was selected according to the best relationship between the initial gelation time and the mechanical properties. The formulations with the highest amount of cross-linking agent (H-5 and H-4) turn out to be the most appropriate due to their initial gelation times and their mechanical resistance. This does not mean that the remaining formulations are not useful in different areas of application.

### 3.2. Initial Gelation Time

**Figure 8:**
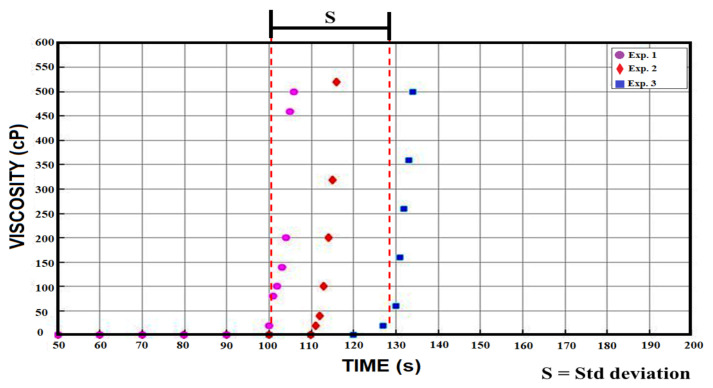
Three experiments of the H-3 formulation are represented in the figure above. Once the 60 cP barrier is exceeded, the viscosity rises exponentially. After a few minutes the photopolymerization is finished and the hydrogel is formed.

**Figure 9:**
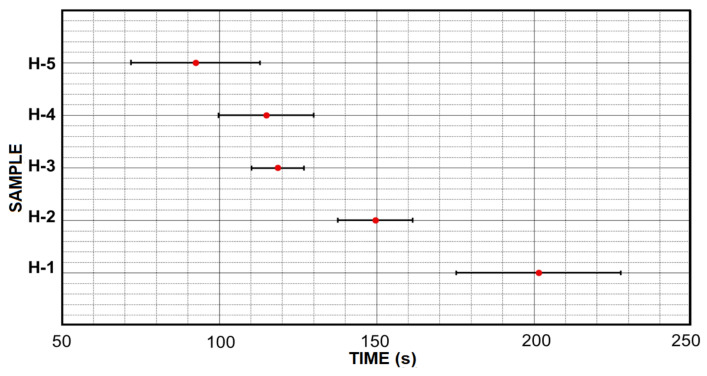
The initiation of gelation times of each sample is shown together with its standard deviation. The H-5, H-4 and H-3 samples show some similarity while the H-1 and H-2 samples are out of phase with their high initiation of gelation time.

Three experiments for each formulation were carried out using a Brookfield viscometer (Figure 8). The initial gelation time is determined as the time where the viscosity of the formulation overpasses 60 cP value. Once the initial gelation time for each formulation is obtained, an average and a standard deviation are calculated for a graphical comparison (Figure 9).

Variation of cross-linking agent (PEGDA) has an effect in the initial gelation time. It is showed in Figure 9 that the sample H-1 has the highest average initial gelation time which is over 200 s. As the amount of cross-linking agent is increased the initial gelation time is reduced, such as the case for the H-2 samples with an average initial gelation time of 150 s. In Figure 9 is observed how H-3, H-4 and H-5 samples share a initial gelation time between 90 and 120 s although it was expected that H-5 formulation would have a shorter initial gelation time compared with the standard (H-3) formulation.

The difference of time shown between the H-5 and H-3 samples is considerably small if we compare the difference shown by the H-1 and H-2 samples, Figure 9. However, the relationship between the concentration of PEGDA and the initial gelation time does not become a linear function. Therefore, it is deduced that the lower amount of cross-linking agent (PEGDA) in the hydrogel formulation, the longer initial gelation time will be obtained. However, a higher concentration of cross-linking agent will not mean a proportional reduction of the initial gelation time. The above behavior can be interpreted as a state of in which a maximum concentration of PEGDA can be used to reduce the initial gelation time. Due to this characteristic to reach our goal in the generation of the material of hydrogel based on PEGDA the most optimal formulations are H-5 and H-4.

### 3.3. Swelling and Cross-Linking Density

**Figure 10:**
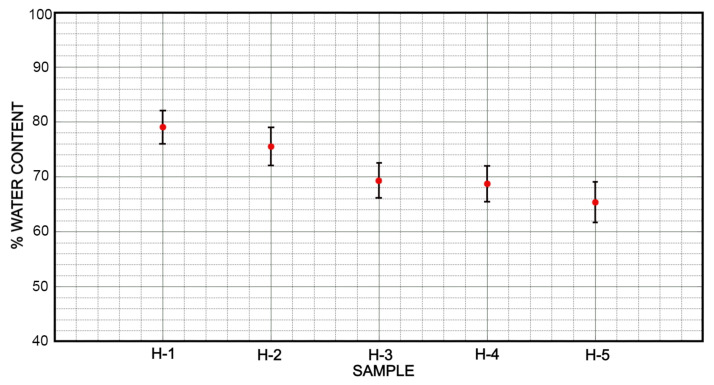
The weight of water content that can be retained by the five hydrogel samples exceeds 60%, however, only the H-1 sample was able to be placed very close to 80% water retention. This water retention also provides them with greater flexibility.

**Figure 11:**
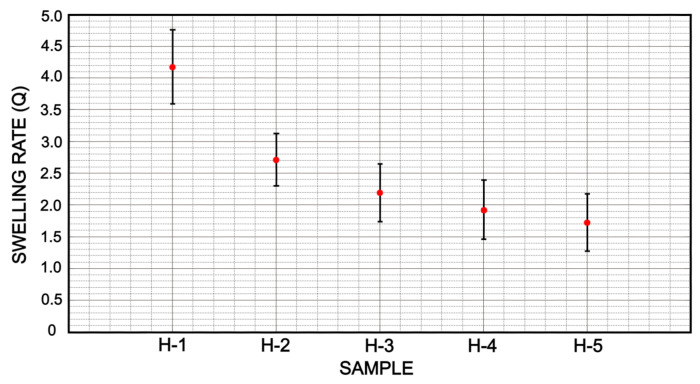
Swelling ratio indicates a greater capacity to retain liquid in hydrogels that have a lower amount of cross-linking agent, such is the case of H-1 and H-2 samples. On the other hand, a higher swelling rate can lead to greater wear of the hydrogel chains and make it less resistant.

The average percentage of water content for hydrogel samples is shown in Figure 10. It is observed that the formulations H-5, H-4 and H-3 have a similar value between 65 and 69% of water content. In the other hand, the formulation H-1 outperforms a greater amount of water content with a value of 79%. The difference in % between formulation H-1 and H-5 is 13.67%. Consequently, the amount of water content is directly related to the proportion of swelling as it is shown in Figure 11. Moreover, a similar relationship is showed in formulations H-5, H-4 and H-3 with respect to their swelling rate. It is observed that the swelling rate of these formulations is up to 4 times their weight, Equation (1). Formulation H-1 is trending upwards by showing a swelling rate over 8 times its weight.

**Figure 12:**
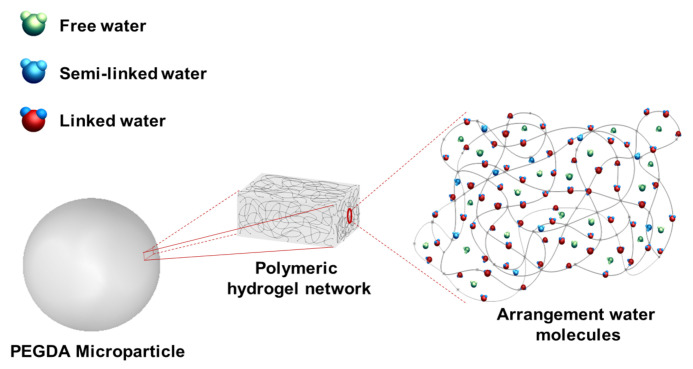
The three physical states of water in a polymer network are shown within the polymeric network of the hydrogel.

The amount of PEGDA used to formulate the hydrogel does influence the amount of water the hydrogel can hold. The water used in the hydrogel formulation can be released almost entirely, however, some of the water remains in the hydrogel structure and it is classified as linked water (Figure 12). Properties of elasticity and flexibility that the hydrogel acquires are due to the amount of semi-linked water inside of it, by hydrogen bridges; this semi-linked water can be forced to be released if the hydrogel is subjected to high shear stress. The free water represents the water that is absorbed into the hydrogel which can be released without the need for high shear stress (Figure 12).

On the other hand, the swelling ratio *Q* shows a variability between formulations H-1 and H-2 which can be useful to achieve a high absorption of water. The formulations H-5, H-4 and H-3 have similar values of *Q*, 1.5, 2.1 and 2.5 (g/g), respectively. Even though these formulations do not reach a large swelling rate, they are still useful for the retention of lower volumes of water.

The swelling results shown above for the different formulations can be explained by the amount of cross-linking agent used in the formulations and the cross-linking degree of the hydrogels obtained. Using the Peppas–Merril model Equation (4) for the swollen networks produced by the cross-linking, it is possible to calculate the average number of molecular weight between two consecutive cross-links ($M_{c}$) (4). Values obtained for each of the formulations are presented in Table 3, and they range between 118 and 196 [g/mol].

**Figure 13:**
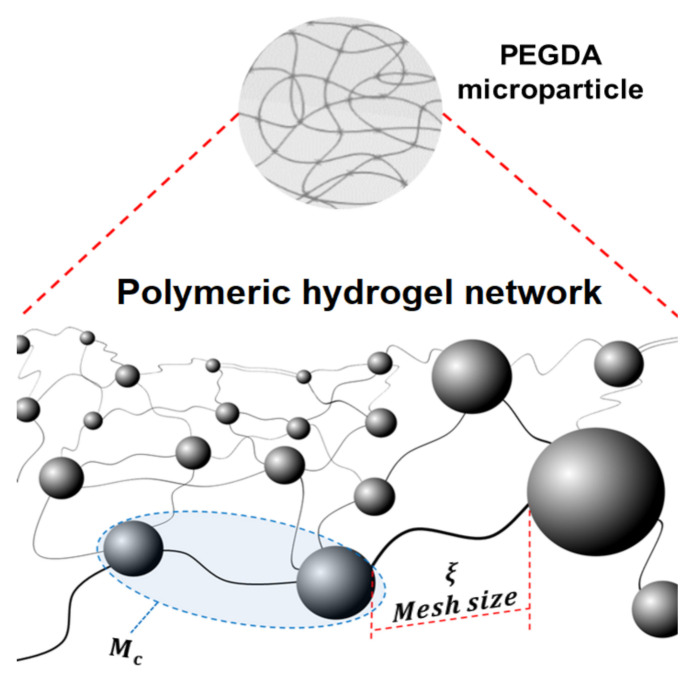
Cross-linked structure of a hydrogel showing mesh size (ξ) and average molecular weight ($M_{c}$). Increasing the concentration of the PEGDA increases the number of acrylate groups which will lead to a formation of higher numbers of free radicals and, therefore, a faster formation of a cross-linked structure, [46,47,48].

The average number of molecular weight between two consecutive cross-links ($M_{c}$) describes the degree of cross-linking between two polymeric chains that intersect to make a connection that constitutes the polymeric network and it influences hydrogel density. The mesh size (ξ) indicates the linear distance between two connected neighboring chains and influences the transfer of solutes passing through the hydrogel, (Figure 13). The values of the parameters $M_{c}$ and ξ are also related to the cross-linking of the hydrogel and the properties that such cross-linking provides to hydrogels. cross-linking in hydrogels influences their elasticity and flexibility, properties linked to the amount of water the hydrogel can absorb. cross-linking network density depends on the conversion of double carbon links between strings, therefore, lower $M_{c}$ values imply the generation of denser cross-linked networks due to a higher formation of double links between strings. As a result, a higher cross-linking density produces a less swelling capacity and flexibility properties. Moreover, higher $M_{c}$ values provide hydrogels with more swelling capacity and flexibility. Due to the cross-linking density it is possible to make different formulations of a hydrogel to obtain the desired properties depending on the area in which the hydrogel is to be implemented.

### 3.4. Soluble Fraction and Drying

**Figure 14:**
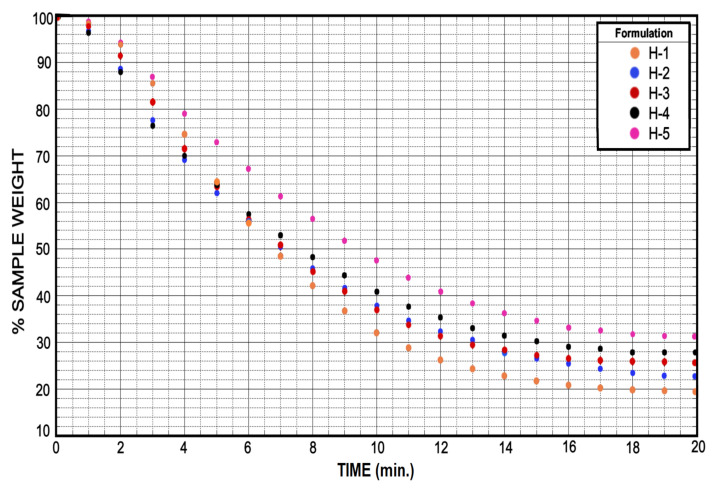
The drying test showed a marked relationship in the behavior of the five samples for the release of the liquid retained in the hydrogel. This guarantees zero interference by the cross-linking agent (PEGDA) in processes involving the discharge of liquid.

Values of dry weight for each of the formulations were averaged and plotted in Figure 14. All formulations have a similar behavior in their drying curve. Each of the endpoints in the Figure 14 corresponds to the percentage (%) weight of the completely dried hydrogel. Therefore, the amount of PEGDA will affect the swelling rate of the hydrogel, but it will not affect the release of water from it. As a result, any of the formulations is useful for the diffusion of aqueous substances facilitating their release or even their absorption in the hydrogels.

**Figure 15:**
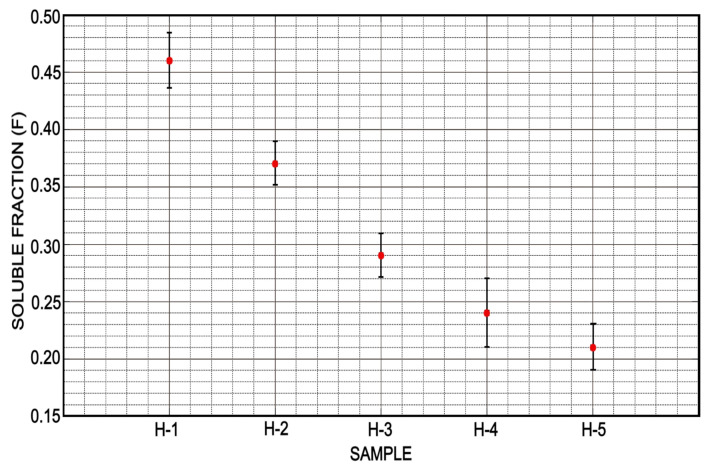
Samples H-3, H-4 and H-5 showed a very similar soluble fraction; however, samples H-1 and H-2 showed a higher value indicating a higher degree of polymer loss caused by the swelling and drying processes, which is counterproductive in desirable reuses.

Solubility of the different formulations is depicted in Figure 15. As it was discussed in the previous section, the formulation H-1, not only has the highest $M_{c}$ value but also the highest soluble fraction with an average value of 0.45 [g/g]. Although the hydrogel formed by the formulation H-1 is capable of absorbing the greatest amount of water, it is also the hydrogel which releases the most “polymeric fraction” when it is deflated. Under conditions where increased water retention in hydrogels is desired, the formulations H-1 and H-2 may work well. However, if it is desired to maintain a certain purity in the absorbed substance when it is released, these formulations could represent some drawbacks. It should be noted that hydrogels based on PEGDA are biocompatible consequently they can be used in biological systems. The hydrogels formed with the formulations H-3, H-4 and H-5 show a lower soluble fraction remaining on average between 0.30 and 0.20 [g/g]. These last three formulations are more suitable if the swelling capacity of hydrogels is to be repeatedly used.

### 3.5. Compression Test and Young’s Module

**Figure 16:**
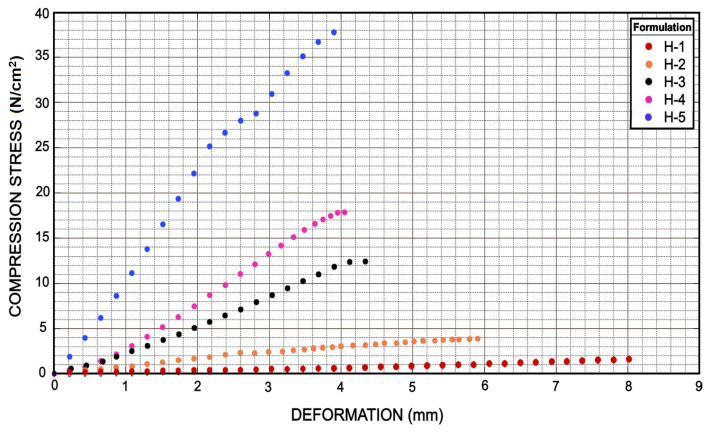
Compress test showed how much hydrogel samples were capable of deformation with respect to the force applied. Clearly H-5 showed a higher strength with a deformation very similar to H-3, while H-1 showed a very low strength in comparison with H-3 as well. However, H-1 was able to double the deformation presented by H-3 and H-5, which translates into greater flexibility.

Compression tests revealed that the addition of a greater amount of PEGDA caused an increase in compression and deformation resistance. It is possible to observe graphically a different behavior for each of the hydrogel samples; Figure 16. Sample H-5 presented the highest compression stress resistance achieving a value of 35.3 N/cm${}^{2}$. Samples H-4 and H-3 appeared to behave similarly by showing compression stress values of 17.9 and 12.5 N/cm${}^{2}$, respectively. On the other hand, sample H-2 showed a value of 3.8 N/cm${}^{2}$ and H-1 showed a value of 1.8 N/cm${}^{2}$ Figure 6; these values were lower than the other samples analyzed. On the other side, Figure 16 also showed the deformation suffered by the samples. Contrary to the compressive stress values, sample H-1 showed a higher deformation value of 8 mm, while sample H-2 showed a deformation value of 5.9 mm. Samples H-3, H-4 and H-5 showed very similar values among them, being 4.36, 4.05 and 3.9 mm, respectively.

**Figure 17:**
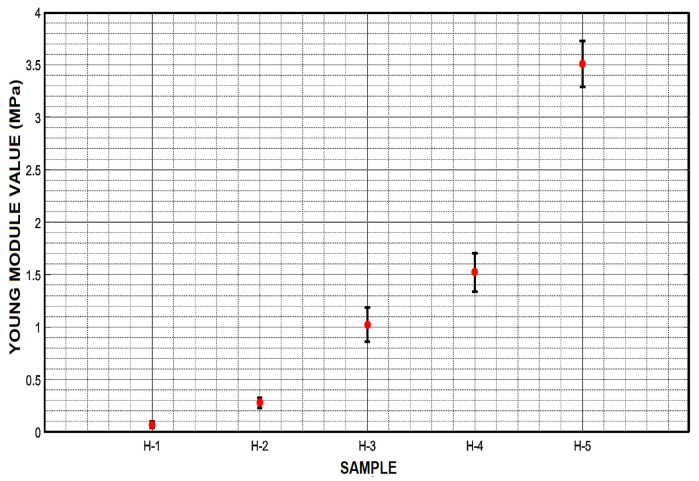
Young’s modulus of the three samples shows a great difference in their ability to behave as an elastic material, being H-1 the most elastic sample and H-5 the strongest sample, triplicate the value of H-3.

Furthermore, the average value of the Young’s module for the highest concentration of cross-linking agent (H-5) was 3.55 ± 0.22 MPa, approximately, which is 3 times higher than the H-3 sample and almost 50 times higher than H-1; Figure 17.

Sample H-3 showed a value of 1.01 ± 0.16 MPa; and it represented significantly a higher value than the H-1 sample, which showed a value of 0.063 MPa, Figure 17.

Sample H-4 presented a Young’s modulus value of 1.52 ± 0.27 MPa while sample H-2 showed a Young’s modulus value of 0.28 ± 0.15 MPa. The Young’s modulus values from the five samples presented in Figure 17 helped us to observe an exponential behavior that is due to the amount of cross-linking agent contained in the hydrogel. The compressive stress values had a similar behavior to the Young’s modulus values.

**Figure 18:**
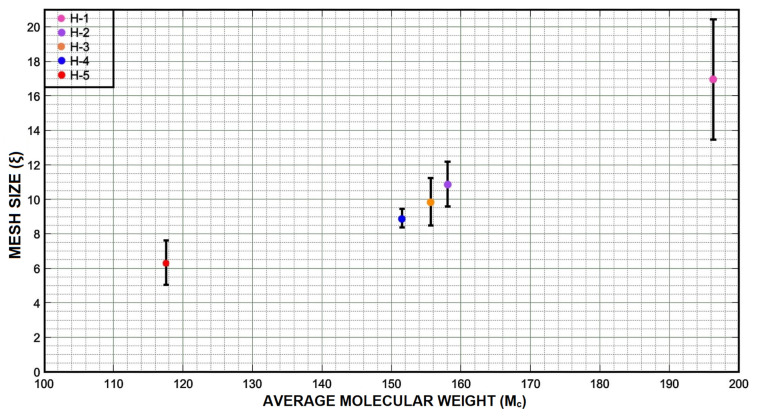
A graphical comparison between $M_{c}$ values and ξ values shows the close relationship that both values have with the amount of cross-linking agent used in different hydrogel formulations.

The difference of the compressive stress value shown by the H-5 formulation compared with the values of the samples H-2 and H-1 was higher. However, a lower compressive stress value is related to a better flexibility in the hydrogel. If we consider the use of hydrogels for the release of medicine or substances into a fluid the formulations, H-1 and H-2 might be more useful. If we take as an example the normal blood pressure (120/80 mmHg) equivalent to 1.6/1.06 N/cm${}^{2}$, the standard formulation might work better than the others [49]. Therefore, if we talk about diffusion, the H-1 formulation could present great advantages since it would have a greater solubility and consequently a greater substance liberation. We can observe the estimated value for each mesh size, ξ, and the average number of molecular weights between two consecutive cross-links, $M_{c}$, of the different samples in Figure 18. Samples H-4, H-3 and H-2 not only showed a similar value of $M_{c}$ but also their mesh size was quite similar. On the other hand, the values of (ξ) shown by hydrogel samples H-5 and H-1 were easily differentiated from the others. The ξ values shown by samples H-5 and H-1 differed from each other by more than 3 Å. In Figure 18, we can see how the values of ξ and $M_{c}$ decrease and increase proportionally to the amount of PEGDA used in each sample. The mesh size values of each sample can be seen in Table 3.

## 4. Conclusions

The experimentation that was carried out in this research demonstrated the importance of the cross-linking agent and its proportion in the formulation. The initial gelation times obtained also showed to be influenced by the amount of cross-linking agent resulting in a shorter initial gelation time at higher concentration of PEGDA. The initial gelation time is expected to be reduced by working with a smaller amount of hydrogel solution inside the microfluidic device. Furthermore, the shortest initial gelation time will allow a use of higher flow rates in the microfluidic devices and in turn allow for a more compact design of them. As a result, the formulation with the shortest initial gelation time, which was formulation H-5 in the present study, will allow the design of smaller and less expensive microfluidic devices.

When a high concentration of PEGDA was used, the polymeric chains of the hydrogel produced were more cross-linked, and therefore, a low $M_{c}$ value was obtained. The mechanical properties studied in this research are showed to be related to the values of $M_{c}$. A hydrogel with a lower $M_{c}$ value will also show a shorter initial gelation time, a lower soluble fraction (*F*), a smaller swelling ratio (*Q*) and, in contrast, a higher resistance to deformation and compression stress. However, a high $M_{c}$ value results in a hydrogel with a good elasticity and flexibility (due to their higher swelling ratio value *Q*) but with a low mechanical resistance. These $M_{c}$ values do not affect the water release capacity of the hydrogel as it was shown in the previous experiments; therefore the hydrogels formed in this research could be possibly used in applications such as a matrix or support for the diffusion of different substances using microfluidic devices.

The analogy obtained between ξ and $M_{c}$ values reinforces the importance of the proportion of cross-linking agent used in the formulation of a hydrogel. Thus a large amount of cross-linking agent will give high mechanical strength to the hydrogel because of its low $M_{c}$ value. On the other hand, a low ξ value will provide the hydrogel with a lower diffusion and in turn a higher protection for agents encapsulated within the hydrogel.

Finally, the knowledge of the $M_{c}$ value and its impact on the mechanical properties of the photopolymerizable hydrogel is of major importance to use in combination with microfluidic devices. The generation of hydrogel-based micromaterials can be applied in multiple fields that require a polymorphic material with specific properties provided by its concentration of cross-linking agent; these fields are such as microparticle formation, encapsulation of microorganisms or release of medicines.

## Figures and Tables

**Table 1:** Volume of the components used for each formulation. The solutions were filled to a volume of 500 mL.

Volume (mL)					
**Substance**	**H-1**	**H-2**	**H-3**	**H-4**	**H-5**
Triethanolamine	018.0	018.0	018.0	018.0	018.0
1-Vinyl-2-pyrrolidinone	002.0	002.0	002.0	002.0	002.0
PEGDA	057.5	086.5	115.0	144.0	172.5
Eosin “Y”	005.0	005.0	005.0	005.0	005.0
Distilled water	417.5	388.5	360.0	331.0	302.5

**Table 2:** Volume of the components used for each formulation. The solutions are filled to a volume of 10 mL.

Volume (mL)					
**Substance**	**H-1**	**H-2**	**H-3**	**H-4**	**H-5**
Triethanolamine	0.36	0.36	0.36	0.36	0.36
1-Vinyl-2-pyrrolidinone	0.04	0.04	0.04	0.04	0.04
PEGDA	1.15	1.73	2.30	2.88	3.45
Eosin “Y”	0.10	0.10	0.10	0.10	0.10
Distilled water	8.35	7.77	7.20	6.62	6.05

**Table 3:** Values used to calculate $M_{c}$ and ξ for each formulation.

$M_{c}$ & ξ Values						
**Formulation**	**Average Q (** $\frac{g}{g}$ **)**	**V** ${}_{2,S}$	**V** ${}_{2,R}$	Mc **Value (** $\frac{\mathrm{gr}}{\mathrm{mol}}$ **)**	**(r** ${}_{0}^{-2}$ **)** ${}^{\frac{1}{2}}$ **(Å)**	ξ **(Å)**
H-1	4.18	0.18	0.12	196.33	8.89	16.93
H-2	2.85	0.24	0.17	158.08	8.03	10.85
H-3	2.50	0.26	0.23	155.70	7.97	09.83
H-4	2.13	0.30	0.29	151.50	7.87	08.88
H-5	1.53	0.37	0.35	117.57	6.90	06.30
